# *Toxoplasma gondii* Infection in Diabetes Mellitus Patients in China: Seroprevalence, Risk Factors, and Case-Control Studies

**DOI:** 10.1155/2018/4723739

**Published:** 2018-12-18

**Authors:** Yong-Xin Li, Hai Xin, Xiang-Yan Zhang, Cui-Ying Wei, Yu-He Duan, Hao-Fu Wang, Hai-Tao Niu

**Affiliations:** ^1^Department of Vascular Surgery, The Affiliated Hospital of Qingdao University, Qingdao, China; ^2^Department of Pathology, The Affiliated Hospital of Qingdao University, Qingdao, China; ^3^Department of Pneumology, The Affiliated Hospital of Qingdao University, Qingdao, China; ^4^Department of Pediatric Surgery, The Affiliated Hospital of Qingdao University, Qingdao, China; ^5^Department of Urinary Surgery, The Affiliated Hospital of Qingdao University, Qingdao, China

## Abstract

The association between* Toxoplasma gondii* (*T. gondii*) infection and diabetes mellitus remains controversial. With the improvement of living standards, the prevalence rate of diabetes is steadily increasing in China. Thus, it is necessary to explore the possible association between toxoplasmosis and diabetes mellitus in China. Hence, case-control studies were conducted to explore the* T. gondii* seroprevalence and identify the risk factors and possible transmission routes of* T. gondii* infection in different types of diabetes, including type 1 diabetes (T1DM), type 2 diabetes (T2DM), and gestational diabetes (GDM) patients in China. Four hundred serum samples for each type of diabetes mellitus, matched with 400 control subjects for each group, were collected and examined for anti-*T. gondii* IgG and IgM antibodies using commercially available enzyme immunoassay kits. The total* T. gondii *seroprevalence in T1DM, T2DM, and GDM patients was 16.50%, 23.50%, and 21.25%, respectively. Each type of diabetes mellitus patients had a significantly higher* T. gondii* seroprevalence than the control subjects. Multivariate regression identified three variables as risk factors for* T. gondii *infection in diabetes patients, including keeping cats at home and consumption of raw oysters for T1DM patients and consumption of raw/undercooked meat and raw oysters for T2DM patients, which may help to guide future research and control policies in diabetes mellitus patients.

## 1. Introduction

*Toxoplasma gondii*, an obligate intracellular opportunistic parasite, can infect nearly all types of warm-blood animals, including humans [1]. Notably, nearly one-third of the human population worldwide have been estimated to be infected with this parasite, and over 7% of Chinese have chronic* T. gondii *infection [1–3]. In general, most* T. gondii* infections do not cause significant clinical symptoms [4]. But in some cases, infected persons may present clinical symptoms of toxoplasmosis such as lymphangoncus, cerebral, and eye diseases [4–6]. In some extreme cases,* T. gondii* infection can be reactivated and lead to a life-threatening disease with involvement of the central nervous system in immunocompromised patients [4, 5].* T. gondii *can reach many organs of the host after infection [7], including the pancreas [8].

Diabetes mellitus is a common chronic metabolic disease and more than 300 million persons worldwide are projected to be affected by this disease in 2030 [9]. With the improvement of living standards, the prevalence rate of diabetes has steadily increased in China. The sensitivity and susceptibility to various infections can be higher in diabetes mellitus patients [10]. In some cases, the Apicomplexan parasite,* T. gondii*, has been proposed as a possible cause of diabetes, and current information is nearly predicated on this issue [11–13]. Meanwhile, chronic toxoplasmosis has been considered as a potential risk factor for type 2 diabetes (T2DM) identified by a meta-analysis of studies on the association between chronic toxoplasmosis and diabetes mellitus [13]. However, type 1 diabetes (T1DM) patients in Colombia were found to have significantly lower* T. gondii* seroprevalence [14]. In USA, no association was found between* T. gondii *infection and diabetes mellitus in a prospective cohort of elderly Latinos [15]. Since data of previous studies on the association between* T. gondii* infection and diabetes mellitus remain controversial, we conducted matched case-control studies to determine whether* T. gondii* seropositivity is associated with different clinical types of diabetes mellitus, including T1DM, T2DM, and gestational diabetes (GDM), and explore the risk factors for* T. gondii *infection in diabetes patients for the first time in China.

## 2. Materials and Methods

### 2.1. Study Sites

The study was conducted in The Affiliated Hospital of Qingdao University, Qingdao (35°35′–37°09′N, 119°30′–121°00′E), Shandong province, eastern China. The Affiliated Hospital of Qingdao University is a large comprehensive hospital in Shandong province that occupies an important position in the national medical system. The patients at this hospital mainly come from five provinces (Shandong, Jiangsu, Liaoning, Jilin, and Heilongjiang).

### 2.2. Study Design and Sample Collection

Through case-control studies, we studied* T. gondii* seroprevalence and identified the risk factors and possible transmission routes of* T. gondii* infection in diabetes mellitus patients and control subjects in China between September 2014 and January 2017. A total of 1200 diabetes mellitus patients who visited the Affiliated Hospital of Qingdao University were included in the study. Three types of diabetes mellitus patients (T1DM, T2DM, and GDM) were invited to participate in this study. The number of patients of each type was 400 (Tables 1–3).

A total of 1200 control subjects, matched with diabetes mellitus patients by age, gender, and residence, were included in the study. Serum samples were obtained from persons who participated in health screening at the Affiliated Hospital of Qingdao University.

Approximately 5 mL of venous blood samples was drawn from participants who gave their consent to participate in this study. Blood samples were left overnight at room temperature to allow clotting and centrifuged at 3000 rpm for 10 minutes. The sera were collected in Eppendorf tubes and stored at 4°C for 24-72 hours and then kept at -20°C until testing.

### 2.3. Sociodemographic, Clinical, and Behavioral Data Collection

Sociodemographic data including age, gender, area of residence, and employment were obtained from all participants. Clinical data including the type of diabetes and behavioral data including obesity and overweight, keeping cats at home, consumption of raw/undercooked meat, fish, oysters, raw vegetables and fruits, gardening or agricultural activities, exposure to soil, source of drinking water, and washing hands before meals were collected from the participants. These variables were selected based on published literature [15, 16]. Data was obtained from the patients/guardians, medical examination records, and informants. Patients were invited to provide veridical information and were informed that their data were used in a confidential manner.

### 2.4. Serological Assay

Sera were analyzed for the presence of IgG and IgM antibodies against* T. gondii *using commercially available enzyme immunoassay kits (Demeditec Diagnostics GmbH, Germany) according to the manufacturer's instructions. Clinical specificity and sensitivity of IgG kit were 99% and 98%, respectively. Clinical specificity and sensitivity of IgM kit were 99% and 100%, respectively [16, 17]. Positive and negative serum controls were included in every plate. To avoid bias of results, the serology test was performed double-blinded. Samples from diabetes mellitus patients and control group were randomly mixed, and the person performing the test did not know the source of samples in advance [16, 17].

### 2.5. Statistical Analysis

Results were analyzed with SPSS 18.0 software package. For the univariate analysis, Chi-square test was used to compare the categorical variables. The Mantel-Haenszel test was used to probe any differences between the patient and control groups. Multivariate regression models were used to adjust for potential confounders. Variables were included in the multivariate analysis if they had a* p* value ≤0.25 in the univariate analysis [17, 18]. Odds ratios (ORs) and the corresponding 95% confidence interval (CI) were calculated, in order to identify the independent risk factors for* T. gondii* infection. Results with a* p* value <0.05 were considered as statistically significant.

### 2.6. Ethics Approval and Consent to Participate

The study protocol was reviewed and approved by the Ethics Committee of the Affiliated Hospital of Qingdao University. The aim of the study was explained to the patients and they provided written consent for their participation in the study. Control sera were collected from volunteers.

## 3. Results

### 3.1. Epidemiology of T1DM Patients with T. gondii Infection

T1DM patients (16.50%) had a significantly higher* T. gondii *seroprevalence than the control subjects (11.50%) (*p*=0.042). Of these, 53 T1DM patients (13.25%) were found to be positive for* T. gondii* IgG antibodies, as compared to 40 controls (10.00%), and the difference was not statistically significant (*p*=0.152).* T. gondii* IgM antibodies were detected in 15 of the 400 T1DM patients and in seven of the 400 controls (3.75%* versus* 1.75%, respectively,* p*=0.084). The details of T1DM patients and control subjects, including age distribution, gender, employment, and area of residence, are shown in Table 1. The highest seroprevalence of* T. gondii* infection was detected in T1DM patients in the age range of ≤30 years (24.19%). T1DM patients living in Shandong province (20.00%) had a higher* T. gondii* seroprevalence than those living in Jilin (14.89%) and Heilongjiang (12.36%) provinces, but the difference was not significant (*p*=0.237). There were no significant differences between female (18.13%) and male (14.98%) T1DM patients (*p* = 0.395). The seroprevalence of* T. gondii* infection among the T1DM patients who lived in rural areas (18.18%) was slightly higher than those who lived in urban areas (14.85%), but the difference was not statistically significant (*p*=0.370). Moreover,* T. gondii* infection seroprevalence was not significantly different among T1DM patients with different types of employment (*p*=0.261).

### 3.2. Epidemiology of T2DM Patients with T. gondii Infection

T2DM patients (23.50%) had a significantly higher* T. gondii *seroprevalence than the control subjects (11.75%) (*p*<0.001). A total of 77 T2DM patients (19.25%) were found to be positive for* T. gondii* IgG antibodies, as compared to 37 controls (9.25%), and the difference was statistically significant (*p*<0.001).* T. gondii* IgM antibodies were detected in 19 of the 400 T2DM patients and in 11 of the 400 controls (4.75%* versus* 2.75%, respectively,* p*=0.137). The details of T2DM patients and control subjects, including age distribution, gender, employment, and area of residence, are shown in Table 2. The highest seroprevalence of* T. gondii* infection was detected in T1DM patients in the age range of 51-60 years (25.81%). T2DM patients living in Shandong province (28.85%) had a higher* T. gondii *seroprevalence than those living in Jilin (22.54%) and Heilongjiang (16.67%) provinces, but the difference was not significant (*p*=0.074). There were no significant differences between male (24.75%) and female (22.22%) T2DM patients (*p* = 0.551). The seroprevalence of* T. gondii *infection among the T2DM patients who lived in urban areas (25.00%) was slightly higher than those who lived in rural areas (22.00%), but the difference was not statistically significant (*p*=0.479). Moreover,* T. gondii* infection seroprevalence was not significantly different among T2DM patients with different types of employment (*p*=0.118).

### 3.3. Epidemiology of GDM Patients with T. gondii Infection

GDM patients (21.25%) had a significantly higher* T. gondii *seroprevalence than the control subjects (12.00%) (*p*=0.042). A total of 70 GDM patients (17.50%) were found to be positive for* T. gondii* IgG antibodies, as compared to 37 controls (9.25%), and the difference was statistically significant (*p*<0.001).* T. gondii* IgM antibodies were detected in 18 of the 400 GDM patients and in 11 of the 400 controls (4.50%* versus* 2.75%, respectively,* p*=0.186). The details of GDM patients and control subjects, including age distribution, gender, employment, and area of residence, are shown in Table 3. The highest seroprevalence of* T. gondii* infection was detected in GDM patients in the age range of >40 years (38.71%), and significant difference was found among different age groups (*p*=0.043). GDM patients living in Shandong province (24.69%) had a higher* T. gondii *seroprevalence than those living in Jilin (12.31%) and Heilongjiang (18.95%) provinces, but the difference was not significant (*p*=0.079). The seroprevalence of* T. gondii *infection among the GDM patients who lived in rural areas (20.20%) was slightly lower than those who lived in urban areas (22.28%), but the difference was not statistically significant (*p*=0.612). Moreover,* T. gondii* seroprevalence was not significantly different among GDM patients with different types of employment (*p*=0.081).

### 3.4. Risk Factors Associated with T. gondii Infection

Univariate analysis showed that some lifestyle variables of T1DM patients had a* p *value ≤0.25, including obesity and overweight, keeping cats at home, consumption of raw/undercooked meat, oysters, and fish, and source of drinking water. Six lifestyle variables of T2DM patients had a* p *value ≤0.25 through univariate analysis. They are obesity and overweight, keeping cats at home, consumption of raw/undercooked meat, fish, oysters, and raw vegetables and fruits. In GDM patients, keeping cats at home and consumption of raw/undercooked meat, fish, and oysters had a* p *value ≤0.25 by univariate analysis. In the multivariate analysis, keeping cats at home (OR=2.885; 95% CI: 1.37-6.07;* p* = 0.005) and consumption of oysters (OR=13.19; 95% CI: 2.91-59.82;* p* = 0.001) were associated with significantly increased odds of* T. gondii* infection in T1DM patients (Table 4). Consumption of raw/undercooked meat (OR=2.663; 95% CI: 1.08-6.56;* p *= 0.033) and oysters (OR=4.785; 95% CI: 1.98-11.45;* p*<0.001) was associated with significantly increased odds of* T. gondii *infection in T2DM patients (Table 4). There was no evidence of a significant association between* T. gondii* status and the selected variables in GDM patients (Table 4).

## 4. Discussion

The association between* T. gondii* infection and diabetes mellitus remains controversial, with few studies reporting conflicting results [19, 20]. Thus, the present study was conducted to determine whether* T. gondii* infection is associated with different types of diabetes mellitus in eastern China. The results showed that diabetes mellitus patients had higher frequencies of antibodies against* T. gondii* as compared to control subjects. Thus, our findings based on serological methods supported an association between diabetes mellitus and* T. gondii* infection.

Type 1 diabetes mellitus (T1DM) is an autoimmune disease with complex interactions between genetic and environmental factors [14]. The enteroviruses and other infectious agents were found to be associated with T1DM [21]. In the present study, T1DM patients had a significantly higher* T. gondii* seroprevalence than the controls (*p*=0.042), suggesting that T1DM patients are more likely to be infected with* T. gondii*. However, further targeted studies should be conducted to explore and confirm the association between T1DM and* T. gondii* infection.

Type 2 diabetes mellitus (T2DM), a major global health problem, is a complex metabolic disease [11]. The incidence of T2DM has notably increased in recent years, in both developed and developing countries [22]. Various infections, including* T. gondii,* may easily appear in T2DM patients because they are immunocompromised [23]. In the present study, T2DM patients had a significantly higher* T. gondii *seroprevalence than the control subjects (*p*<0.001). These evidences indicated a potential association between* T. gondii *infection and T2DM implying that* T. gondii *infection may increase susceptibility to T2DM, while T2DM patients are more vulnerable to opportunistic infections such as* T. gondii*. Importantly, the clinician should pay more attention to* T. gondii* infection when they diagnose and treat T2DM patients given the high prevalence of* T. gondii* infection in T2DM patients, and* T. gondii* infection may aggravate the T2DM status.

*T. gondii* infection during pregnancy may cause serious consequences such as miscarriage, microcephaly, hydrocephalus, and severe neurological disorders in the fetus [5]. In addition, in immunodeficient individuals, released bradyzoites from tissue cysts switching back into rapidly multiplying tachyzoites could cause reactivation of latent infection and dissemination throughout the body [24]. The immune system in diabetes mellitus patients is affected, and GDM patients are more susceptible to* T. gondii* infection. In the present study, GDM patients had a significantly higher* T. gondii* seroprevalence than the control subjects (p<0.001). Thus, serological screening of GDM patients is needed, followed by proper treatment of the* T. gondii* infection [25]. Moreover, information about toxoplasmosis and its transmission routes should be given to GDM patients as part of prenatal care.

The first epidemiological investigation on* T. gondii* infection in humans in China was conducted in Guangxi Zhuang Autonomous Region in 1978 [26]. Now, toxoplasmosis has become a notifiable disease in China. However, there are no national guidelines for the prevention of toxoplasmosis in China. Humans acquire the infection through three major routes: consumption of undercooked meat containing* T. gondii* tissue cysts, ingesting oocysts-contaminated water, soil, vegetables, and fruits, and transmission from mother to fetus during pregnancy [1, 27]. As expected, we found that keeping cats at home and consumption of raw/undercooked meat were associated with significantly increased odds of* T. gondii* infection in diabetic patients. These two risk factors have been identified in many studies in China [16, 28–30]. Interestingly, fresh oyster consumption was also a potential risk factor for* T. gondii* infection in T1DM and T2DM patients, which was similar to a study reported from the United States [31].* T. gondii* oocysts can be washed into the sea via rainwash and runoff [32, 33], and shellfish including oysters, clams, and mussels can ingest the oocysts directly from seawater [1, 32–37]. In China,* T. gondii *oocysts have been detected in oysters [38] and consumption of fresh oysters is common in recent years, which may explain the higher* T. gondii *seroprevalence in the diabetes mellitus patients who consume raw oysters than those who do not consume raw oysters. Thus, knowledge of these risk factors will help in prevention efforts.

Some limitations of the present study should be kept in mind. First, our study participants might not represent the general clinically healthy individuals, pregnant women, and diabetes mellitus population due to the potential limitation of enrollment methods. Therefore, potential selection bias should be considered when interpreting our results. Second, serology could not clearly indicate the infection status as current infection or past infection; potential bias caused by such misclassification could not be eliminated. Moreover, molecular identification, taxonomy, genetic variation, and diagnosis of* T. gondii* should be considered in further studies. Third, more effective statistical analysis methods should be used to confirm the association between diabetes mellitus and* T. gondii* infection. Therefore, our results need to be proved in further studies.

## 5. Conclusion

This study provided serological evidence of an association between* T. gondii* infection and three types of diabetes mellitus (T1DM, T2DM, and GDM). Moreover, keeping cats at home and consumption of raw/undercooked meat and raw oysters were risk factors for* T. gondii* positivity using multivariate regression, which may help to guide future research and control policies. Further studies should be conducted to elucidate the role of* T. gondii* in diabetes mellitus.

## Figures and Tables

**Table 1:** Seroprevalence of *T. gondii* infection by sociodemographic factors in type 1 diabetes (T1DM) patients and controls in eastern China.

Characteristic	T1DM (N=400)				Controls (N=400)				T1DM *vs.*
	Prevalence of *T. gondii* infection				Prevalence of *T. gondii* infection				Controls
	No. tested	No. positive	%	*P-*value	No. tested	No. positive	%	*P-*value	*P-*value
Age									
≤ 30	62	15	24.19	0.187	62	10	16.12	0.591	0.263
31-40	70	12	17.14		85	12	14.12		0.604
41-50	100	13	13.00		90	8	8.89		0.367
51-60	92	18	19.57		86	8	9.30		0.053
>60	76	8	10.53		67	8	11.94		0.789
Region									
Shandong	170	34	20.00	0.237	170	23	13.53	0.493	0.110
Jilin	141	21	14.89		141	13	9.22		0.144
Heilongjiang	89	11	12.36		89	10	11.24		0.816
Gender									
Male	207	31	14.98	0.395	221	23	10.41	0.447	0.155
Female	193	35	18.13		179	23	12.85		0.160
Area of residence									
Urban	202	30	14.85	0.370	211	16	7.85	0.031	0.019
Rural	198	36	18.18		189	27	14.29		0.299
Employment									
Unemployed	156	28	17.95	0.261	137	21	15.33	0.147	0.549
Employed part-time	139	26	18.71		161	13	8.07		0.006
Employed full-time	105	12	11.43		102	12	11.76		0.940
Obesity and overweight									
Yes	156	21	13.46	0.191	194	24	12.37	0.596	0.762
No	244	45	18.44		206	22	10.68		0.021
Keeping cats at home									
Yes	50	16	32.00	0.002	58	10	17.24	0.138	0.074
No	350	50	14.29		342	36	10.53		0.134
Consumption of raw/undercooked meat									
Yes	26	8	30.77	0.043	60	10	16.67	0.174	0.140
No	374	58	15.51		340	36	10.59		0.052
Consumption of oyster									
Raw	94	32	34.04	<0.001	134	16	11.94	0.845	<0.001
Boiled	306	34	11.11		266	30	11.28		0.950
Consumption of fish									
Raw	147	34	23.13	0.007	167	19	11.38	0.948	0.006
Boiled	253	32	12.65		233	27	11.59		0.721
Consumption of raw vegetables and fruits									
Yes	204	37	18.14	0.368	228	27	11.84	0.805	0.066
No	196	29	14.80		172	19	11.05		0.287
Exposure to soil									
Yes	232	40	17.24	0.639	247	32	12.96	0.246	0.190
No	168	26	15.48		153	14	9.15		0.182
Gardening or agricultural activities									
Yes	257	42	16.34	0.909	257	33	12.84	0.260	0.261
No	143	24	16.78		143	13	9.09		0.053
Washing hands before meals									
Yes	262	44	16.79	0.827	266	28	10.53	0.390	0.036
No	138	22	15.94		134	18	13.43		0.559
Source of drinking water									
Spring/well	85	19	22.35	0.101	68	13	19.12	0.031	0.625
Tap	315	47	14.92		332	33	9.94		0.054
Total	400	66	16.50		400	46	11.50		0.042

**Table 2:** Seroprevalence of *T. gondii* infection by sociodemographic factors in type 2 diabetes (T2DM) patients and controls in eastern China.

Characteristic	T2DM (N=400)				Controls (N=400)				T2DM *vs.*
	Prevalence of *T. gondii* infection				Prevalence of *T. gondii* infection				Controls
	No. tested	No. positive	%	*P-*value	No. tested	No. positive	%	*P-*value	*P-*value
Age									
≤ 30	37	9	24.32	0.956	37	4	10.81	0.793	0.127
31-40	83	19	22.89		87	11	12.64		0.080
41-50	96	23	23.96		99	13	13.13		0.052
51-60	93	24	25.81		97	12	12.37		0.018
>60	91	19	20.88		90	7	7.78		0.012
Region									
Shandong	156	45	28.85	0.074	156	25	16.03	0.025	0.007
Jilin	142	32	22.54		142	17	11.97		0.019
Heilongjiang	102	17	16.67		102	5	4.90		0.007
Gender									
Male	202	50	24.75	0.551	211	27	12.80	0.492	0.002
Female	198	44	22.22		189	20	10.58		0.002
Area of residence									
Urban	200	50	25.00	0.479	229	18	7.86	0.005	<0.001
Rural	200	44	22.00		171	29	16.96		0.223
Employment									
Unemployed	163	43	26.38	0.118	169	24	14.20	0.326	0.006
Employed part-time	133	23	17.29		148	13	8.78		0.033
Employed full-time	104	28	26.92		83	10	12.05		0.012
Obesity and overweight									
Yes	152	30	19.74	0.152	190	20	10.53	0.470	0.017
No	248	64	25.81		210	27	12.86		<0.001
Keeping cats at home									
Yes	52	19	36.54	0.017	56	9	16.07	0.279	0.015
No	348	75	21.55		344	38	11.05		<0.001
Consumption of raw/undercooked meat									
Yes	28	14	50.00	<0.001	161	5	3.11	<0.001	<0.001
No	372	80	21.51		339	42	12.39		0.001
Consumption of oyster									
Raw	91	41	45.05	<0.001	143	15	10.49	0.559	<0.001
Boiled	309	53	17.15		257	32	12.45		0.119
Consumption of fish									
Raw	150	50	33.33	<0.001	167	22	13.17	0.454	<0.001
Boiled	250	44	17.60		233	25	10.73		0.031
Consumption of raw vegetables and fruits									
Yes	253	66	26.09	0.109	286	32	11.19	0.581	<0.001
No	147	28	19.05		114	15	13.16		0.203
Exposure to soil									
Yes	228	54	23.68	0.920	248	34	13.71	0.120	0.005
No	172	40	23.26		152	13	8.55		<0.001
Gardening or agricultural activities									
Yes	248	56	22.58	0.580	254	35	13.78	0.096	0.011
No	152	38	25.00		146	12	8.22		<0.001
Washing hands before meals									
Yes	270	63	23.33	0.910	285	30	10.53	0.232	<0.001
No	130	31	23.85		115	17	14.78		0.075
Source of drinking water									
Spring/well	90	20	22.22	0.745	64	13	20.31	0.020	0.776
Tap	310	74	23.87		336	34	10.12		<0.001
Total	400	94	23.50		400	47	11.75		<0.001

**Table 3:** Seroprevalence of *T. gondii* infection by sociodemographic factors in gestational diabetes (GDM) patients and controls in eastern China.

Characteristic	GDM (N=400)				Controls (N=400)				GDM *vs.*
	Prevalence of *T. gondii* infection				Prevalence of *T. gondii* infection				Controls
	No. tested	No. positive	%	*P-*value	No. tested	No. positive	%	*P-*value	*P-*value
Age									
≤ 30	185	35	18.92	0.043	201	26	12.94	0.017	0.107
31-40	184	38	20.65		71	19	26.76		0.294
>40	31	12	38.71		28	3	10.71		0.014
Region									
Shandong	239	59	24.69	0.079	239	32	13.39	0.451	0.002
Jilin	65	8	12.31		65	8	12.31		1.00
Heilongjiang	95	18	18.95		95	8	8.42		0.035
Area of residence									
Urban	202	45	22.28	0.612	245	25	10.20	0.165	0.001
Rural	198	40	20.20		155	23	14.84		0.192
Employment									
Unemployed	126	24	19.05	0.081	98	15	15.31	0.470	0.464
Employed part-time	143	39	27.27		197	20	10.15		<0.001
Employed full-time	131	22	16.79		105	13	12.38		0.343
Obesity and overweight									
Yes	79	15	18.99	0.582	117	9	7.69	0.086	0.018
No	321	70	21.81		283	39	13.78		0.011
Keeping cats at home									
Yes	40	12	30.00	0.135	47	4	8.51	0.433	0.010
No	360	73	20.28		353	44	12.46		0.005
Consumption of raw/undercooked meat									
Yes	33	12	36.36	0.027	77	5	6.49	0.098	<0.001
No	367	73	19.89		323	43	13.31		0.021
Consumption of oyster									
Raw	84	30	35.71	<0.001	153	18	11.76	0.909	<0.001
Boiled	316	55	17.41		247	30	12.15		0.084
Consumption of fish									
Raw	137	42	30.66	<0.001	158	17	10.76	0.086	<0.001
Boiled	263	43	16.35		242	41	16.94		0.858
Consumption of raw vegetables and fruits									
Yes	282	64	22.70	0.275	299	37	12.37	0.692	0.001
No	118	21	17.80		101	11	10.89		0.149
Exposure to soil									
Yes	233	47	20.17	0.534	254	34	13.39	0.261	0.045
No	167	38	22.75		146	14	9.59		0.002
Gardening or agricultural activities									
Yes	257	52	20.23	0.505	263	35	13.31	0.265	0.034
No	143	33	23.08		137	13	9.49		0.002
Washing hands before meals									
Yes	266	54	20.30	0.513	302	38	12.58	0.529	0.013
No	134	31	23.13		98	10	10.20		0.011
Source of drinking water									
Spring/well	78	14	17.95	0.840	66	15	22.73	0.003	0.476
Tap	322	61	18.94		334	33	9.88		<0.001
Total	400	85	21.25		400	48	12.00		<0.001

**Table 4:** Multivariate analysis of selected characteristics of diabetic patients and their association with *Toxoplasma gondii* infection.

Type of diabetic patients	Characteristic^a^	Adjusted Odds ratio^b^	95% Confidence interval	*P* value
T1DM	Obesity and overweight Keeping cats at home	0.584 2.885	0.32-1.08 1.37-6.07	0.086 0.005
	Consumption of raw/undercooked meat Consumption of oyster	2.177 13.19	0.81-5.88 2.91-59.82	0.125 0.001
	Consumption of fish	0.295	0.06-1.36	0.117
	Source of drinking water	1.713	0.78-3.77	0.181
T2DM	Obesity and overweight Keeping cats at home Consumption of raw/undercooked meat	0.673 1.584 2.663	0.39-1.15 0.79-3.17 1.08-6.56	0.147 0.193 0.033
	Consumption of oyster	4.758	1.98-11.45	<0.001
	Consumption of fish	0.747	0.32-1.72	0.493
	Consumption of raw vegetables and fruits	1.296	0.72-2.33	0.385
GDM	Keeping cats at home Consumption of raw/undercooked meat	1.376 1.906	0.63-2.99 0.83-4.36	0.420 0.127
	Consumption of oyster	1.764	0.78-3.99	0.173
	Consumption of fish	1.516	0.72-3.18	0.271

^a^The variables with a *p *<0.25 in the univariate analysis were included.

^b^Adjusted by age and the other characteristics included in this table.

## Data Availability

The clinical and behavioral data used to support the findings of this study are included within the article.
